# A novel application of lemmatize and thematic analysis to understand protective measures perspectives of patients with chronic respiratory disease during the first outbreak of COVID-19 pandemic: a qualitative study

**DOI:** 10.3389/fpubh.2024.1351754

**Published:** 2024-08-29

**Authors:** Domingo Palacios-Ceña, David Peña-Otero, Ciro Casanova-Macario, Juan Nicolas Cuenca-Zaldivar, Cristina Garcia-Bravo, Cesar Fernandez-de-las-Peñas, David Díaz-Pérez

**Affiliations:** ^1^Research Group of Humanities and Qualitative Research in Health Science of Universidad Rey Juan Carlos (Hum&QRinHS), Department of Physical Therapy, Occupational Therapy, Physical Medicine and Rehabilitation, Universidad Rey Juan Carlos, Alcorcón, Spain; ^2^Research Group of Nursing in Instituto de Investigación Sanitaria Valdecilla (IDIVAL), Santander, Spain; ^3^Department of Nursing, Hospital Universitario Marqués de Valdecilla, Servicio Cántabro de Salud, Santander, Spain; ^4^Department of Pulmonology, Hospital Universitario Nuestra Señora de Candelaria, Servicio Canario de la Salud, Santa Cruz de Tenerife, Spain; ^5^Universidad de Alcalá, Facultad de Medicina y Ciencias de la Salud, Departamento de Enfermería y Fisioterapia, Grupo de Investigación en Fisioterapia y Dolor, Alcalá de Henares, Spain; ^6^Research Group in Nursing and Health Care, Puerta de Hierro Health Research Institute-Segovia de Arana (IDIPHISA), Majadahonda, Spain; ^7^Research Group of Evaluation and Assessment of Ability, Functionality and Disability of Universidad Rey Juan Carlos (TO+IDI), Department of Physical Therapy, Occupational Therapy, Physical Medicine and Rehabilitation, Universidad Rey Juan Carlos, Alcorcón, Spain; ^8^Research Group in Manual Therapy, Dry Needling and Therapeutic Exercise of the Universidad Rey Juan Carlos (TMPSE), Department of Physical Therapy, Occupational Therapy, Physical Medicine and Rehabilitation, Universidad Rey Juan Carlos, Alcorcón, Spain; ^9^Nursing Care Research Department, Servicio Canario de la Salud, Tenerife, Spain

**Keywords:** chronic obstructive pulmonary disease, COVID-19, SARS-CoV-2, life change events, life course perspective, qualitative research

## Abstract

**Objective:**

To describe the perspectives of a group of COPD patients during the first outbreak of the COVID-19 pandemic and narrate the emotions and polarity (acceptance-rejection) regarding living with COPD during the pandemic.

**Design/methods:**

We used a novel application of lemmatization and thematic analysis of participants’ narratives. A study was carried out with eight patients with moderate–severe-very severe COPD during the first outbreak of COVID-19 using purposive sampling. In-depth interviews and field notes from the researchers were used to collect data. A statistical content analysis (lemmatization) of the patients’ narratives was performed. Additionally, inductive thematic analysis was used to identify emerging themes. This study was conducted following the guidelines of Consolidated Criteria/Standards for Reporting Qualitative Research. The study was conducted in accordance with the principles articulated in the WMA Declaration of Helsinki. Participants provided verbal informed consent prior to their inclusion as previously described.

**Results:**

The average age of our sample was 65 years, and 75% of the patients suffered from moderate COPD, 12.5% from severe COPD, and 12.5% from very severe COPD according to GOLD criteria. The lemmatized and sentiment analysis showed a predominance of positive emotions, and the polarity of the interviews indicated a very slight positive trend towards acceptance of the experience lived during the pandemic. Additionally, three main themes were identified: (1) Confinement and restriction measures, (2) COVID-19 and protective measures, and (3) Clinical care during the first outbreak of the pandemic.

**Conclusion:**

Patients experienced confinement with a feeling of security and protection. They strictly respect social distancing. They used masks, but these caused them to feel short of breath and fatigue, especially FFP2 masks. Half of the patients rejected the possibility of being vaccinated. Finally, they were very satisfied with the clinical care they received in the COPD unit of their hospital. Our results show that COPD patients have not experienced a negative impact of the COVID-19 pandemic.

## 1 Introduction

To date, patients with chronic obstructive pulmonary disease (COPD) have a fourfold in-creased risk of developing severe forms (1) and are more likely to be affected by COVID-19 (2). Patients with COPD are known to be more susceptible to respiratory viral infection and virus-induced exacerbations caused by influenza, rhinovirus, and seasonal coronaviruses (3, 4). The incidence of hospitalization and severity of illness in patients with COPD are much higher in patients with COVID-19 than in those with seasonal influenza (5). Awatade et al. (3) in their systematic review show that people with COPD do not have a higher prevalence of hospital admission. Therefore, this could indicate that there is no increased susceptibility to SARS-CoV-2 infection or it may reflect a change in community behavior – which benefits from social isolation. Also the implementation of infection control measures during the COVID-19 pandemic has reduced the number of respiratory infections, which is the most common cause of COPD exacerbations (6).

Despite this, studies show that the number of COPD patients admitted at hospital for SARS-CoV-2 infection ranges from 2 to 7.7% and in intensive care units, this prevalence does not exceed 5% (7, 8). These results may be due to the protective effect of inhaled drugs, due to the underdiagnosis of COPD, which reaches 74.7% in Spain (9), and the strict compliance with isolation measures (10). The “lockdown” that the worldwide population has been forced to endure during the first COVID-19 outbreak has led to the emergence of a situation of psychosocial instability that may have worsened due to the mass quarantine (11). These aspects have several consequences in the natural history of the patient’s disease, with high psychiatric morbidities such as depression and anxiety.

Therefore, efforts should be made to maintain a situation of low clinical impact over time in individuals with COPD, since clinical management in COPD is an integrative, dynamic and useful tool (12) where the patient’s perception is key to their treatment. In Italy, feelings of terror, fear and/or apprehension were reported in 58.22% of COPD patients during the first months of the pandemic (13). The result of COPD patients suffering from COVID-19 is that the patient reports increased shortness of breath and worse quality of life and sleep, combined with mood disturbances. However, previous studies had shown how the “lockdown” and confinement had a low impact on COPD patients, albeit many clinical consultations and tests were cancelled, patients were very satisfied with the telephonic care provided (14).

During the COVID-19 pandemic it has been shown how COPD patients experienced an increase in their feeling of fragility (15) and, faced with the fear of becoming infected and dying (6), patients adopted restrictive protective measures (16, 17), by changing their perception of risk (17). As a consequence, there was an increase in protective measures against contagion, but also a decrease in social contact and isolation in their homes (10, 16, 17). New technologies and virtual platforms and chats helped to maintain contact with professionals and continue with COPD treatment (18, 19).

Therefore, the aims of this study were: (a) to describe the perspectives of a group of Spanish patients with COPD during the first outbreak of Covid-19 pandemic regarding confinement, restrictive measures, protective strategies adopted, and clinical care during the pandemic, and (b) to narrate the emotions and polarity (acceptance-rejection) of their perspectives regarding living with COPD during the pandemic applying a novel lemmatize and thematic analysis of participants’narratives.

## 2 Materials and methods

### 2.1 Design

A qualitative descriptive case study was conducted (20) based on constructivist paradigm (21). Case study is a research proposal that explores or describes a single case bounded in time and place (e.g., individuals in pandemic period). Also, case studies are suitable for answering how and why research questions or can be used to describe patient perspectives or experiences regarding care (22, 23). The Consolidated Criteria for Reporting Qualitative Research (24) (Supplementary Table S1) and the Standards for Reporting Qualitative Research guidelines (25) (Supplementary Table S2) were used. Qualitative studies have been used to research the barriers and facilitators to COPD rehabilitation, the expectations before and after COPD treatment and the use of technology to follow-up COPD treatment (26–28).

Seven researchers (two women), including one pulmonary and lung specialist doctor, three nurses, two physical therapists and one occupational therapist participated in this study, three of whom (DPC, CGB, CFP) had experience in qualitative study designs. Three researchers (DDP, CCM, and DPO) had clinical experience in COPD. Prior to the study, the positioning of the researchers was established according to their previous experience and their motivation.

### 2.2 Participants and sampling strategies

In this study, a non-probabilistic, purposeful sampling strategy was used based on relevance to the research question rather than representativeness (29). Participants were recruited from the high complexity COPD unit of the Complejo Hospitalario Universitario de Canarias belonging to the CHAIN (COPD History Assessment in Spain) cohort (30).

The inclusion criteria were: (a) Patients >18 years of age, (b) diagnosed with COPD of at least 12 months of evolution according to the GesEPOC criteria (31), presenting a moderate (GOLD 2)—severe (GOLD 3) -very severe (GOLD 4) stage according to the Global Initiative for Chronic Obstructive Lung Disease (GOLD) criteria (32); an FEV1/FVC ratio of less than 0.7 after bronchodilator testing and who also have an airflow obstruction of less than 80% of the theoretical FEV1, and (c) with internet connection and owning a device capable of making video calls. The exclusion criteria were: (a) patients with cognitive impairment, and/or with verbal communication disorders, (b) presenting auditory or verbal sensory disorders preventing proper communication with the interviewer.

In qualitative research, a wide variety of proposals exist for justifying and determining sample size (33, 34). Furthermore, there is no formula for the prior calculation of the sample size (33, 34). Due to the unavailability of many cases (lockdown), the sample size was determined following Pragmatic Considerations (34), and the Information Power criteria proposed by Malterud et al. (35). In the present study, all available cases were included in order to obtain a greater richness of the data. In such scene, information power indicates that the more information relevant for the current study the sample holds, the lower number of participants is needed (35). The sample size with enough information power (and fewer number of participants) depends on: (a) a specific project objective aimed at the analysis of a phenomenon to be studied (narrow study); (b) the specificity of the sample, with a homogeneous and defined profile of the participants (dense sample); and (c) the application of a specific analysis strategy to specific and defined cases (participants), using different analysis strategies (35). The present study meets the above criteria by having objectives focused on the perspectives of patients with a specific type of disease (COPD), with a certain degree of severity (moderate–severe-very severe), under specific conditions (first outbreak of the COVID-19 pandemic) and using various analysis systems (lemmatization and thematic analysis of the interviews) (36).

### 2.3 Data collection

Semi-structured interviews including open questions were used to obtain information regarding the issues of interest (29). After collecting professional and personal data from each participant, the broad opening question was: “Please, can you share with me your personal experience during the COVID-19 pandemic?” Open-ended follow-up questions were used to obtain detailed descriptions (Table 1). Additionally, “Please tell me more about that,” was also used to enhance the depth of the discussion of a specific topic. Thereafter, the researchers noted the key words and topics identified in the patients’ responses and used their answers to ask further questions and to clarify the content (29).

**Table 1:** Question guide used for the semi-structured interviews.

Areas of research	Questions
Disease	What is it like to live with moderate/severe COPD? What is more relevant to you? What are your expectations about the disease and its evolution? What was it like living with COPD during the first wave of the COVID-19 pandemic?
Treatment during the COVID-19 pandemic	What is more relevant to you about COPD treatment during the pandemic? Have you made any changes to adapt to the preventive measures against COVID-19?
The COVID-19 pandemic and expectations concerning the patient’s health status	How had the pandemic influenced your health status and the evolution of your disease? What has been the most relevant for you? How did you experience the risk of infection with COVID-19? What strategies do you use to avoid infection? How did you experience confinement during the first wave of the pandemic in Spain?
The COVID-19 pandemic and its relationship with health professionals and/or professional help-seeking	How would you describe your relationship with healthcare professionals? How had the COVID-19 pandemic influenced your relationship with healthcare professionals?
The COVID-19 pandemic and barriers and facilitators to professional help-seeking in healthcare facilities	What barriers or facilitators had you perceived during the COVID-19 pandemic to seeking professional help and/or accessing a healthcare facility? What is the most relevant to you?
Confinement during the pandemic	How did you experience the confinement during the pandemic? What was the most relevant for you? How do you think the confinement had influenced your health? Could you describe what your emotions and feelings were during the outbreak?
Family relations	How did the pandemic affect your family life and your relationships with each other?
Vaccine	How do you feel about the vaccine? What barriers or facilitators have you encountered in getting the vaccine?

Due to the lockdown situation for flattening the COVID-19 curve established by the Spanish Government on 14th March 2020 interviews were conducted in a private video chat room using the Microsoft Teams videoconference platform^1^ (37). Each participant received a private/personalized email with an invitation. Table 2 shows the specific procedure followed for the interviews using zoom platform.

**Table 2:** Interview procedure using Microsoft Teams (https://www.microsoft.com/es-ww/microsoft-teams/log-in).

At the prearranged date and time, the participant and researcher both clicked on the Microsoft Teams link and entered into the private video chat room.
The interview involved the researcher first sharing the screen with the participant and reviewing an informed consent form together, reading and ensuring participant comprehension.
After verbal consent to participate was provided, all participants were offered an email copy of the consent form.
The researcher asked for participants´ permissions for recording the interview in both video and audio, and after confirming the participant’s consent, the researcher turned on the recording. If a participant declined to record the video, only audio was recorded.
The researcher opened the semi-structured interview guide (in Microsoft Word document) on his/her computer and started the interview.
The researcher asked participants to describe their experiences, perspectives and feelings during the COVID-19 outbreak, to obtain a better understanding of how their unique situation may affect their comprehension or interpretation of its interview.
During the interview, the researcher took notes on the participant’s responses. At the end of the interview, and when the patient’s considered it suitable, the audio/video recording was stopped.

All interviews were audio- and video-recorded after oral permission was granted by the participants, in order to access non-verbal cues such as eye contact, facial expressions or body motions, which are unique data resources for qualitative studies. Videorecording enabled the collection as much non-verbal information as possible, which could enrich the descriptions of participants’ experiences. All interviews were transcribed verbatim, recording a total of 427 min of interviews over-all (average of 53.37, SD 10 min). The interviews were managed by CGB, DPO, and JNCZ.

Furthermore, field notes were collected by the researchers during the semi-structured interviews since they provide a rich source of information as participants describe their personal experiences, their behaviors during data collection, and enable researchers to note their reflections concerning methodological aspects of the data collection (36).

Sociodemographic data were collected from the participants (age, sex, whether they were an active smoker, marital status, job, and who they lived with) and data from the Chronic Obstructive Pulmonary Disease Assessment Test-CAT scale (38), and the BODE index (39). The COPD Assessment Test o CAT, assessing the impact of COPD on health status, is a short and simple instrument consisting of eight items covering disease symptoms and restricted activity (38). Also, the BODE Index is a multidimensional scoring system that has been developed as a prognostic marker for COPD patients and integrates the respiratory and the systemic expressions of COPD. It is composed of body mass (B), degree of airflow obstruction (O), level of functional dyspnea (D) and exercise capacity (E) (40). Confidentiality was assured by consecutively numbering each interview and removing identifying information from the transcripts. All audio recordings and transcripts were saved on a password-protected computer with restricted access only by the researchers.

### 2.4 Analysis

The interviews were analyzed by means of a lemmatize textual content analysis of the participants’ words and narratives (41), and an inductive thematic analysis for the identification of the relevant themes obtained from the interviews (29, 36).

From the lemmatize textual content analysis we obtained: (a) a cloud of the most used words, and (b) an identification of the feelings of the participants and the polarity (acceptance or rejection) of their narratives. The use of lemmatize textual content analysis in interviews and written texts through statistical techniques is used in discourse analysis and qualitative studies as a method of deepening and triangulating the analysis (36). Also, the statistical analysis of narratives and transcribed interview texts in qualitative studies has been previously used in studies on understanding COPD disease through patient narratives (42), analysis of electronic health records (43), and death in intensive care unit (44).

#### 2.4.1 Lemmatize textual content analysis

For the lemmatize textual statistical analysis of the qualitative content (36), the software R version 3.5.1 (R Foundation for Statistical Computing, Institute for Statistics and Mathematics, Welthandelsplatz 1, 1020 Vienna, Austria) was used. The text of the interviews was lemmatised for analysis. A word frequency analysis was carried out using the tf-idf algorithm (term frequency—inverse document frequency), and a word-cloud, representing the most frequent use of words within the participants’ interviews, was obtained. Emotion analysis was performed using Bing (45), Afinn (46) and National Research Council Canada (NRC) dictionaries (41, 47). Text polarity was analyzed, using the SODictionaries V1.11Spa dictionary as amplifiers and decrementators, the Bing dictionary (45), and as deniers, those proposed by Vilares et al. (48). For the analysis of polarity (acceptance-rejection) four stages were used. In the first stage, a file was created with the text from the interviews, broken down by sentences for textual analysis. In the second stage, polarity was calculated using the Bing Sentiment Dictionary, amplifiers and de-amplifiers of SODictionar-iesV1.11Spa (41, 47) and the deniers proposed by Vilares et al. (48) (Supplementary Table S3). In the third stage, the scatter diagram of the sentences in the text in relation to neutrality was calculated to identify positive or negative tendencies. Finally, the evolution of emotional valence (positive–negative) was shown throughout the interviews. Fourier transformation was applied to confirm the polarity trend.

#### 2.4.2 Thematic analysis

**Figure 1:**
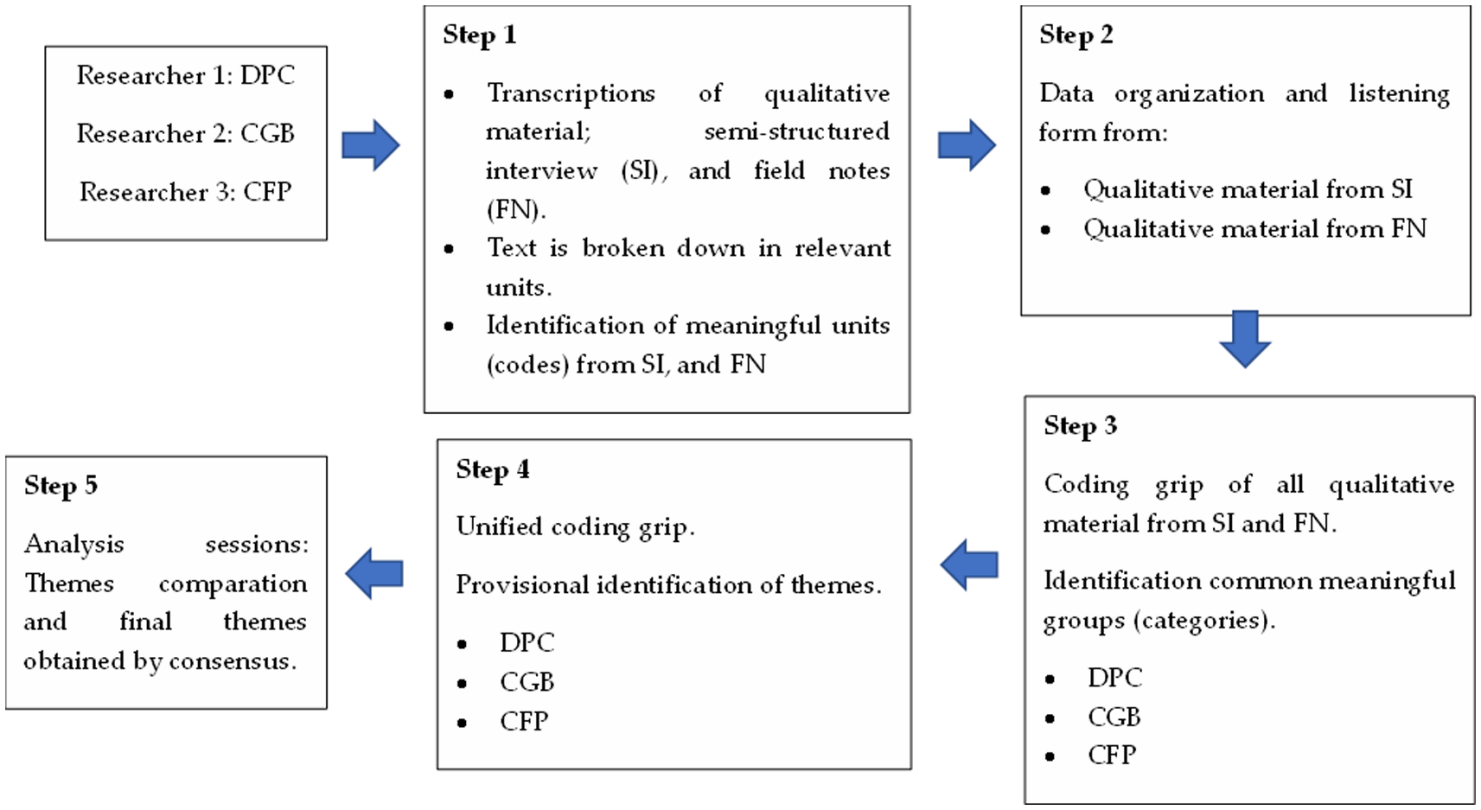
Description of the data analysis process.

Full transcripts were made of each semi-structured interview and of the researchers’ field notes. Inductive analysis (36) consisted of identifying text fragments with relevant information to answer the research question. From these narratives, the most descriptive contents (codes) were identified. Subsequently, these units were grouped by their common meaning (categories) and/or similar content. Thematic analysis was applied separately to interviews and field notes by DPC, CGB, and CFP. Joint team meetings were held to combine the results of the analysis and discuss data collection and analysis procedures. In these team meetings, the final themes were displayed, combined, integrated and identified. In case of divergence of opinions, the identification of the theme was based on consensus among the members of the research team. Finally, three themes were identified (Figure 1).

### 2.5 Rigor and trustworthiness

We used criteria by Guba and Lincoln for establishing trustworthiness of the data (29). Table 3 summarizes the procedures used to enhance trustworthiness.

**Table 3:** Trustworthiness techniques.

Criteria	Techniques performed and application procedures
Credibility^1^	Investigator triangulation: each interview was analyzed by three researchers. Thereafter, team meetings were performed in which the analyses were compared, and themes were identified.
	Triangulation of data collection methods: semi-structured interviews were conducted and researcher field notes were kept.
	Triangulation of analysis methods: inductive thematic analysis and content analysis of narratives were used.
	Member checking: this consisted of asking the participants to confirm the data obtained during the data collection.
Transferability^2^	In-depth descriptions of the study were performed, providing details of the characteristics of researchers, participants, contexts, sampling strategies, and the data collection and analysis procedures.
Dependability^3^	Audit by an external researcher: an external researcher assessed the study research protocol, focusing on aspects concerning the methods applied and study design.
Confirmability^4^	Investigator triangulation, data collection and analysis triangulation.
	Researcher reflexivity was encouraged via the completion of reflexive reports and by describing the rationale for the study.

1 Credibility confidence in the truth of the findings.

2 Transferability reporting that the findings have applicability in other contexts.

3 Dependability reporting that the findings are consistent and could be repeated.

4 Confirmability the degree to which findings are determined by the respondents and not by the biases, motivations, and interests of researchers (29).

### 2.6 Ethical considerations

This study was approved by the Clinical Research Ethics Committee of the Complejo Hospitalario Universitario de Canarias (Canary Islands Health Service, Spain; code: CHUNSC_2020_79-October 15, 2020). The study was conducted in accordance with the principles articulated in the WMA Declaration of Helsinki. Participants provided verbal informed consent prior to their inclusion as previously described.

## 3 Results

The sample of the present study consisted of eight patients with COPD (five women) with a mean age of 65.12 years (SD 6.87). Six patients had moderate COPD, one had severe COPD, and one had very severe COPD. Two participants continued to smoke (P1 and P7), the number of packs of tobacco per year that they continued to consume were 45, and 35, respectively for P1 and P7. The characteristics of the participants are shown in Table 4.

**Table 4:** Sociodemographic and clinical features of patients.

Patients	Age	Sex	CAT scale^1^	BODE index	Active smoker	Civil status	Work status	Living with
P1	71	Female	13	1	Yes	Divorced	Permanent incapacity for work	Lives with relatives
P2	67	Female	13	6	No	Married	House wife	Lives with partner
P3	74	Male	9	0	No	Married	Retired	Lives with partner
P4	71	Male	1	0	No	Married	Retired	Lives with partner
P5	62	Male	4	0	No	Divorced	Retired	Lives with partner
P6	55	Female	8	2	No	Divorced	Actively working	Lives with partner
P7	64	Female	11	1	Yes	Divorced	Actively working	Lives alone
P8	57	Female	15	7	No	Divorced	Sick leave. Inability to work/study	Lives alone

1 CAT: COPD (chronic obstructive pulmonary disease) assessment test.

### 3.1 Results of lemmatized textual content analysis

**Figure 2:**
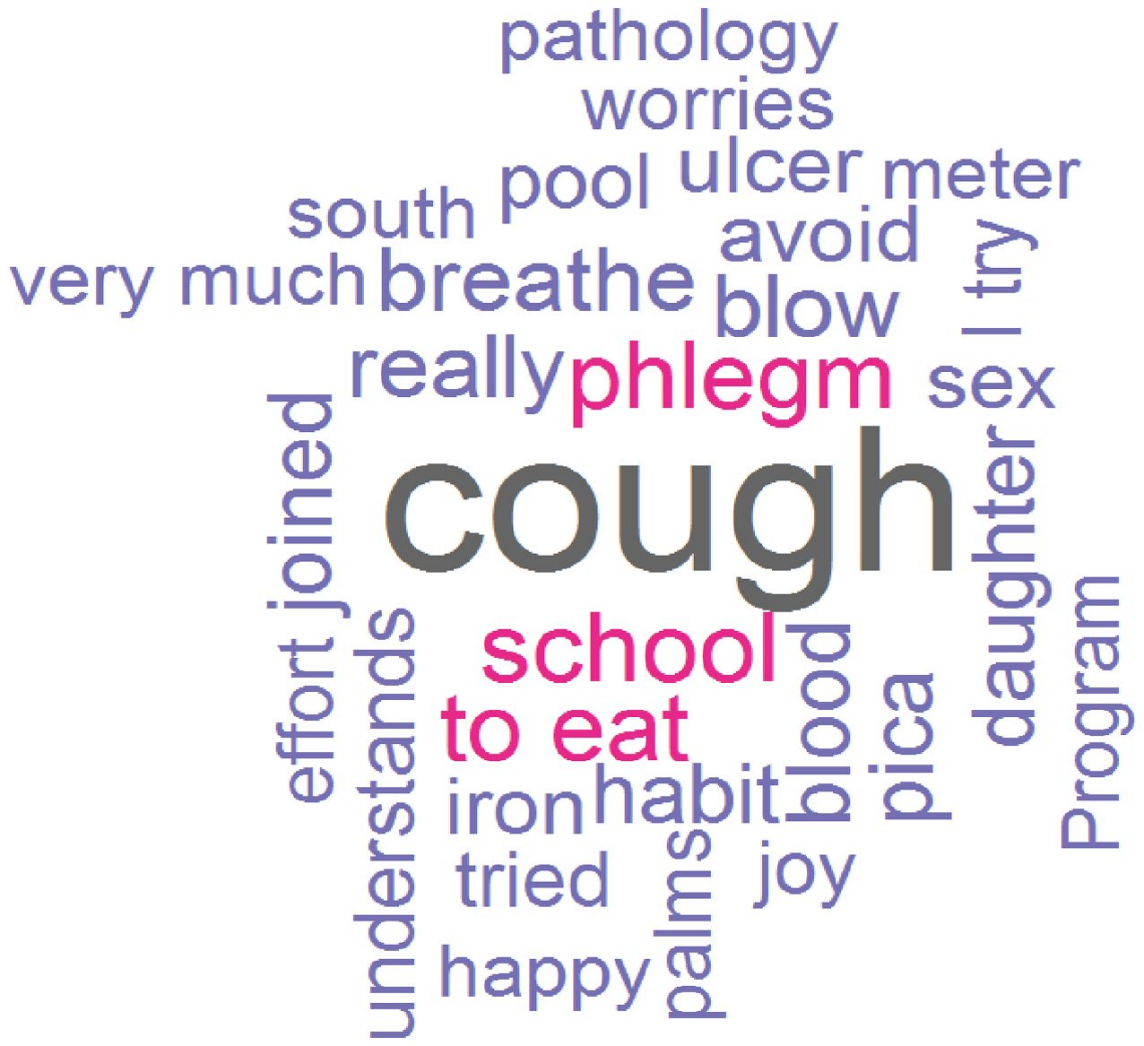
Word cloud.

**Figure 3:**
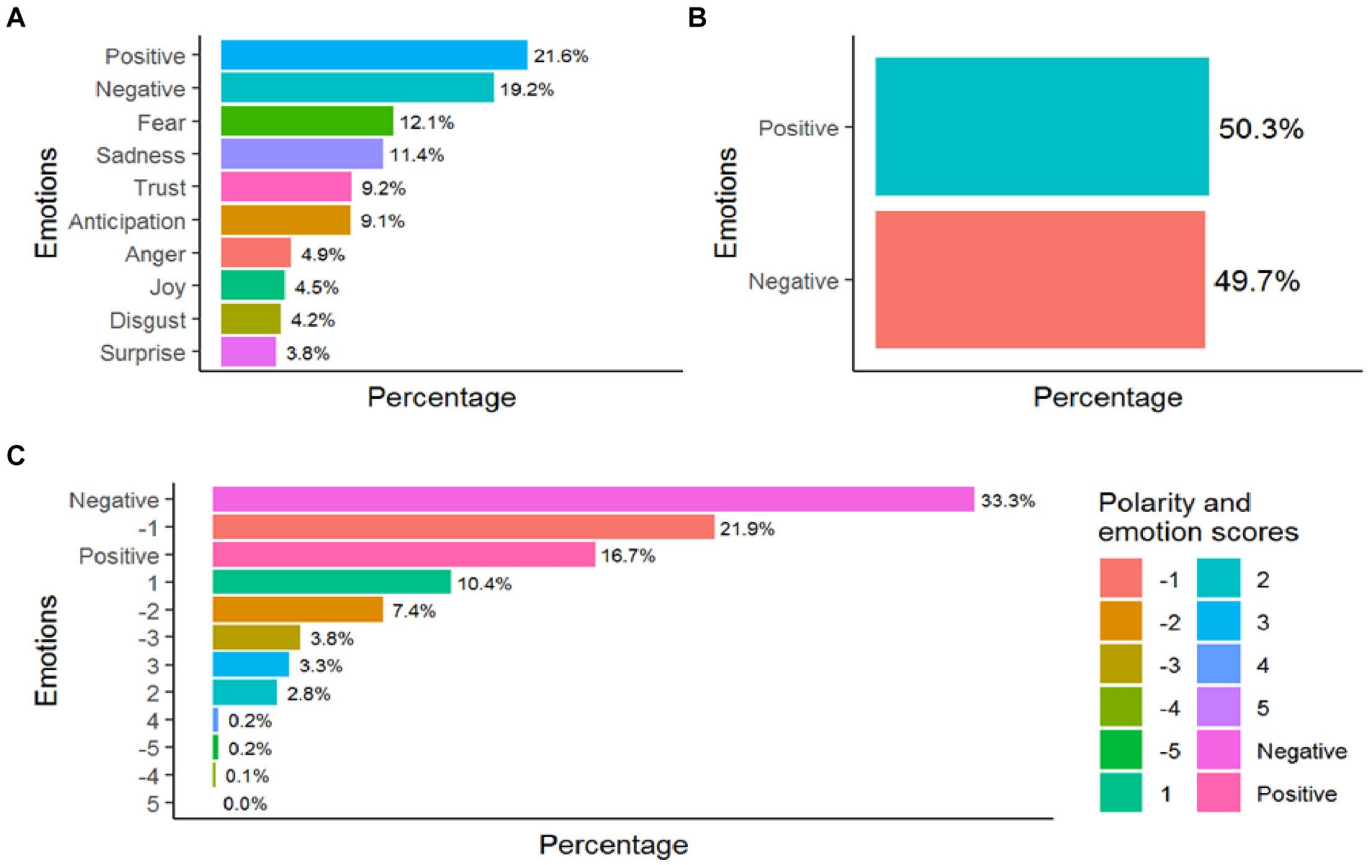
Sentiment analysis **(A)** from NRC dictionary, **(B)** from Bing dictionary, and **(C)** from Afinn dictionary.

The word cloud showed how cough, school and phlegm are the most repeated followed by to eat, blow, breathe, and blood (Figure 2). The sentiment analysis showed a predominance of positive emotions (Figure 3) from NRC (Figure 3A), and Bing (Figure 3B) dictionaries sentiment scores. In the case of the Afinn dictionary there is a predominance of negative emotions, especially scores −1 and − 2 against positive scores of 1 (Figure 3C). The associated emotions are those of fear and sadness, followed by trust and emotions of anticipation (Figure 3A).

**Figure 4:**
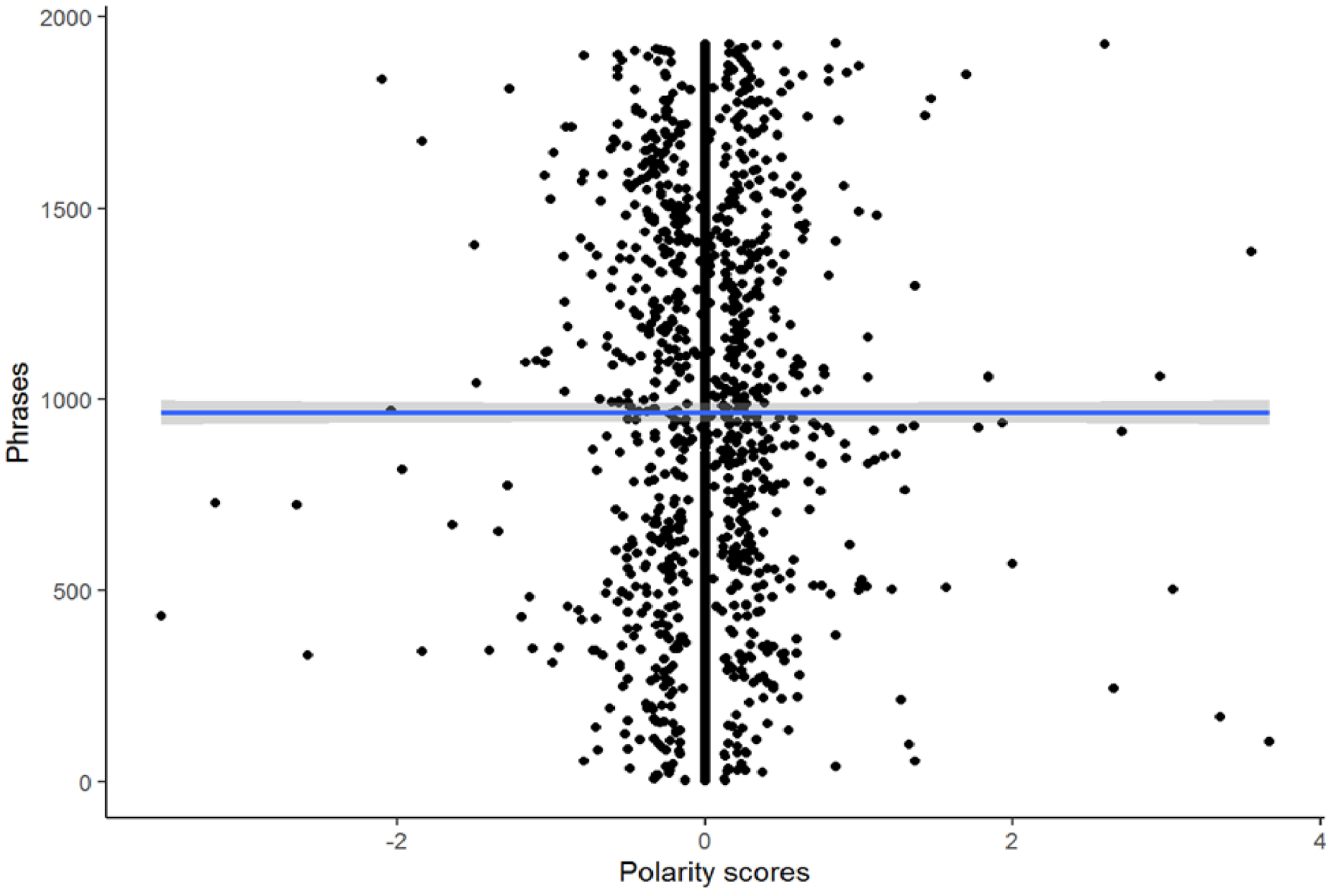
Polarity analysis.

The polarity of the interviews is 0.01 ± 0.421 which indicates a very slight positive trend towards positive emotions due to the presence of some more extreme positive values (Figure 4).

### 3.2 Results of the thematic analysis

Three main themes were identified: (1) Confinement and restrictive measures, (2) COVID-19 and protective measures, and (3) Clinical care during first pandemic out-break. Participants’ narratives, extracted directly from interviews, described each identified theme.

#### 3.2.1 Theme 1: confinement and restrictive measures

All participants reported that the confinement did not affect their daily life or their disease. Most participants reported that before the pandemic, COPD already caused symptoms (fatigue, shortness of breath) that limited their activity, and they had to modify their habits to a greater or lesser degree. They adapted their lives to remain at home most of the day. Thus, the confinement did not entail a substantial change in their life routine:

“Throughout all the pandemic, I was at home, I was doing my business and I have not noticed any change. I have a bed, I have a fridge, I have a computer and I spend the day there” (P2).

All the participants reported that during the pandemic they limited their trips out of the house and the time they spent outdoors. They were more wary of going out and when they did, they complied with all the protective measures, masks, hand hygiene, and social distancing.

In addition, most participants described how they endured the confinement with positive feelings, perceiving it as a protective measure, as they were more at-risk, they felt more protected and safer:

“I saw it as protection for me. I had no bad feelings. It’ s true that there were moments of anguish for not being able to go out at any time, but I did not have bad feelings. My feelings were more positive than negative. At the end, confinement, for people with my disease, was a protective measure. That was how I experienced it” (P6).

Indeed, some participants described that the pandemic has heightened their awareness and concern about the evolution of COPD, and the need to follow clinical recommendations. In the case of participant 8, the confinement even helped her COPD:

“During the confinement I improved. I was able to train and exercise gently and progressively every day. Before the pandemic I had 24% lung capacity, and I finished the confinement with 37.7% lung capacity” (P8).

The participants reported that the pandemic did not trigger conflicts in their relationships with their families and partners. On the contrary, it made the family more protective, more vigilant and closer. This resulted in them carrying out all those tasks that could be a risk of contagion for the patient (going shopping, going to the pharmacy, etc.). Moreover, the family decreased the frequency of visits, and the number of people visiting the patients:

“I'll give you an example of how some things have changed. On Christmas Eve we used to gather up to 50 people at my house for dinner and now my wife and I have dinner alone. The whole family agrees, they all protect us…” (P3).

The participants referred that their social relationships have changed. They have become more distant. One participant recounted how she perceives more distance and feels lonely, even when going to the doctor:

“Above all, the change has been the feeling of loneliness. Before COVID-19, when you went to the doctor, you were always talking to people, everyone was sitting around talking to someone. Now no one talks to anyone, you go into the health center and wait, you are really on your own, with your illness” (P2).

Some participants described how the pandemic has changed their relationship with the society, with people, but has not changed their life or way of living with COPD.

“With the pandemic, what has changed the most in me is my relationship with other people. My way of living my life remains the same, with my illness. But with people it is different, more distant, socially distant” (P4).

#### 3.2.2 Theme 2: COVID-19 and protective measures

Some participants reported feeling like a target, as they are more vulnerable to COVID-19 and being considered at risk. This feeling was continuous and was generated from different sources, i.e., the media, family, health professionals, etc.

All participants except one (P4) reported being afraid. Fear of contagion, fear of being vulnerable, fear of their COPD and/or their lungs getting worse because COVID-19, fear of getting sick and not having the opportunity to say goodbye to their family, fear of intubation and being admitted into the ICU. Some patients reported that their fear was due to having COPD. If they were not at so much risk of dying because of their COPD they would not be afraid:

“If I did not have COPD, I would not be so afraid, I would, but it would not be like it is now. Having COPD is one more thing that means that if I get COVID-19, it will harm me more and I would have a higher risk of die. I have an affected lung and if I get COVID-19 I have a greater risk of dying. That’s what I fear” (P3).

Participants described how they avoided seeking clinical help and going to the primary care center, because despite the online appointment-based care systems, at the health center there were many people together, without social distancing, waiting to be seen in the same room. Another measure they adopted was to stay at home, avoid going out, if they went for a walk, they would cross to a side of the street that was empty, avoid crowds, and above all avoid approaching and encountering people. They recounted how the use of gloves, hydroalcoholic gel, and face masks intensified as the pandemic progressed.

“I came to avoid going to the medical center when I felt bad or that I was breathing worse. There you were with many people who did not follow the rules and the feeling of risk of contagion was very strong. “I even changed sidewalk on the street, trying to be alone, with no one around” (P3).

In relation to masks, only one participant reported that they adapted without difficulty (P4). The rest of the participants confirmed that the use of face masks made them more tired, experiencing greater fatigue when walking, feelings of breathlessness and shortness of breath:

“I am uncomfortable. It’ s a mask, which prevents me from breathing, because of my COPD, I breathe even worse, and I feel even more breathless. So, I must take it off” (P2).

They had the feeling that they are in a prison, they noticed that the air they breathe is not “pure,” experiencing feelings of choking and suffocating. One of the participants even described how, due to the use of the face mask, their COPD can worsen due to the fact that they are continuously breathing air that was high in CO2:

“I left my house without a face mask. I hate it, it's something that's harming me. If I have COPD, I'm swallowing the same air that I'm expelling. I think that hurts me, because the air is not purified, all the carbon dioxide goes inside. And so that is suffocating me a lot” (P3).

Participants noted that the face mask that most accentuates these sensations is the FFP2 mask (equivalent to other international standards known as N95, KN95 and P2 masks). They were aware that it is the face mask that protects them the most, yet, it is the one that makes them feel the most distressed when they go out. Conversely, they explained that this sensation of suffocation and fatigue decreases with the surgical mask:

“When I go to a closed place and I must put on the FFP2, I get anxious, I have a terrible time, I have to stop every three steps to breathe and lower the face mask. With the surgical mask, it's more bearable, at least you can breathe better, you don't get tired so much, it filters the air more” (P2).

Most participants reported that they usually need to lower or remove the mask to continue walking. They end up with accelerated breathing and pulsations due to the effort:

“I can wear the mask, but with enormous effort and I arrive with my breathing totally accelerated and my heart beating at full speed [makes the sound and gesture of beating]” (P6).

Regarding the use of vaccines, half of the participants were in favor of their use, but the other half rejected them. The reasons they gave for not wishing to be vaccinated were distrust of their rapid development, distrust of the pharmaceutical industry, and lack of experience. They pointed out that the use of information about the vaccine and the organization of vaccination programs by politicians has not helped. Among the participants who did not accept the vaccine, some were likely to change their minds once time has passed and the vaccine has been tested first in other people, and it is confirmed that there are no significant risks or negative effects. The theory used to justify their refuse to vaccination is based on beliefs of how COVID-19 affects the lung, if the vaccine contains part of the virus, people with COPD should not get the vaccine as there would be more risk of that part of the vaccine virus attacking their already damaged lungs:

“The vaccine can affect my lung, just like COPD. If I am going to treat a lung disease and the virus attacks the lung, and they are going to give me a vaccine to make it easier for the virus to go to the lung… Then, farewell… I wouldn't get vaccinated even with a gun to my head” (P3).

All participants, those who were likely to accept or refuse vaccination against COVID-19, justified their decision using the flu vaccine as a comparison. Thus, those who accepted the vaccine stated that the COVID-19 vaccine is much like the flu vaccine, considering that it is safe and should be given periodically in people at higher risk of complications, such as COPD patients. Meanwhile, those who rejected it argued that the COVID-19 vaccine is unlike the flu vaccine, stating (from their perspective) that the vaccine introduces the virus and goes straight to the lung, whereas the flu vaccine does not attack the lung and is safer. Both groups of participants accepted the flu vaccine.

“The flu vaccine is different. It has been tested for a long time, and it is safe. In addition, the virus does not get inside you to attack your lungs. “The flu vaccine protects your entire body” (P4).

#### 3.2.3 Theme 3: clinical care during the first outbreak of the pandemic

Participants described how the main changes they experienced have been in primary care, where access to clinical consultations has become more difficult, due to the suspension of face-to-face care, the delay of appointments, and the implementation of an online appointment request system, and telephone care, which limited participants’ face-to-face access to the doctor:

“The first barrier is getting an appointment, everything online, when you get it, it's over the phone, no contact, and in the end if they decide you don't need to be seen you don't get anyone in person to assess you. You assume that what they tell you over the phone will work” (P4).

Subsequently, the physician assesses whether the participants require face-to-face care. However, even when a face-to-face consultation is carried out, the professional distance is maintained. For some of the participants who need to see a primary care physician and wish to be evaluated by a professional, it is a difficult decision because they perceived it as a risk of contagion:

“You want to go to the doctor like before, to have contact, to be touched, to be examined, but on the other hand you are afraid, because of the risk of contagion, it is a closed place, a lot of people waiting, all together” (P1).

In contrast, participants reported that the specialized care provided by the hospital’s COPD unit remained the same, with face-to-face consultations, scheduled appointments, and periodic follow-up and monitoring of treatment and the disease as if there were no pandemic:

“As for my COPD, I have not noticed any change in the monitoring they do. Throughout the pandemic they have continued to keep an eye on my tests, on when I have to do the next one, on the control of my disease, on everything. Everything has remained the same with or without a pandemic” (P1).

The participants reported that it makes them feel safe and protected, because there is a group of professionals who, despite the pandemic, are in charge of monitoring the disease and controlling its progress:

“I feel fortunate, because they are taking care of me, whether there is a pandemic or not, they have continued to be there, monitoring me so that my COPD does not continue. I feel very much safeguarded with them, I was afraid that they would stop monitoring COPD patients” (P5).

All concurred that they have a very close relationship with the professionals in the unit, and that their experience has been very positive. The reasons reported by participants are having close contact with the same staff for a long time, face-to-face care, maintenance of the follow-up and surveillance protocol during the pandemic and resolving doubts and incidents regarding COPD and COVID-19.

### 3.3 Integration of thematic analysis and results for emotions and polarity

In the thematic analysis, patients with COPD described how confinement did not affect their level of activity and showed how it helped protecting them, but fear of contagion increased the adoption of protective measures (wearing masks and avoiding close contact with people). Content analysis of emotions and polarity showed fear as the main emotion identified, along with a negative trend in the narrative of the participant’s speech. On the other hand, the results of the thematic analysis showed important negative aspects (fears of contagion, dissatisfaction with medical care, etc.). However, when determining the polarity (acceptance/rejection) of the discourse about the pandemic and the impact on their lifes, the final result was positive. The identified words focused on describing the COPD clinic but were not related to the COVID-19 pandemic.

## 4 Discussion

Our thematic results showed that patients with COPD did not have a major negative experience of confinement during the first wave of COVID-19 outbreak, following the recommendations to avoid the infection. They had many difficulties with face masks, particularly FFP2 type, and half of them refused the vaccine. Ultimately, they feel safe and protected by the health care professionals in the COPD unit of their hospital.

Upon triangulation of the thematic results with textual content analysis, participants showed a positive polarity of their discourse regarding the first wave of the COVID-19 pandemic. Our results coincided with the work of Pleguezuelos et al. (14) on the experience of patients with COPD during their confinement in the COVID-19 pandemic, where they described how most of the patients stated that their feeling regarding lung disease and their general health was similar or even better during confinement, and how it had little impact on their life. This contrasts with previous studies of COPD patients during the pandemic, which described negative feelings, along with anxiety, and stress (10, 16, 49). The psychological and emotional impact described in previous studies due to the pandemic were a consequence of the fear of dying, fear of becoming infected, feeling abandoned by the health system, difficulty of access to health professionals, and lack of support and care, despite being vulnerable patients (10, 15, 16, 49). The positive polarity of our participants’ perspective, identified in their narratives, can be explained by the fact that the patients belong to a specialized COPD cohort/unit of a regional hospital, where specific treatment and follow-up protocols were in place, where the Referring professionals (physicians and nurses) have not changed, and where care protocols have continued to be applied in the same way as before the pandemic. Previous studies have shown how the involvement and proactivity of professionals minimized the adverse effects of the COVID-19 pandemic on patients with COPD (10, 18, 19).

Regarding home confinement, our participants did not experience it in a negative or distressing way. On the contrary, they recounted how these measures were particularly beneficial to vulnerable patients with chronic conditions. These results agree with an observational study (14) conducted in Spain, where 100 COPD patients were interviewed by telephone. The Hospital Anxiety and

Depression, the COPD Assessment Test, and the 5-Dimension Euro Quality-of-Life questionnaire were administered. The interviews also included questions about the lockdown, missed clinical

appointments, and fears of the disease. In this study, Pleguezuelos et al. (14) showed that, in general, the worldwide lockdown had a low impact on COPD patients. In contrast, in Italy, during the

confinement, COPD patients described feeling terror, anguish, and apprehension (13). This difference compared to our results could be explained by the fact that the restrictive measures were applied to

all people, decreasing the exposure of COPD patients to other non-vulnerable people, and therefore decreasing the risk of contagion. Moreover, prior to the pandemic, patients had adapted their habits and lifestyle to the restrictions caused by COPD symptoms. Whereas Philip et al. (49) described the concerns and difficulties of patients with COPD regarding obtaining food, money, supplements, and medication, keeping their distance from their social and family environment, and in those living alone, in our results these difficulties were not reported because they were performed by the partner, or the family (in the case of those who lived alone).

Our results showed how patients with COPD felt more vulnerable, and they strictly adopted protective measures (avoiding going to the doctor, using masks and gloves, hydroalcoholic gel, etc.) due to fear (of contagion, of COPD progressing and worsening, of intubation). Moreover, agreed with previous studies (10, 16) that reported how patients with COPD during the pandemic, due to the fear of becoming infected, have implemented strict measures of social distancing, self-isolation, and distancing from family and friends. Measures such as wearing face masks, social distancing, washing or sanitizing hands, and avoiding public or crowded places, were largely followed and applied and in a higher proportion by patients with chronic diseases compared to the healthy population (50). However, Kusk et al. (16) described how patients with COPD, by applying strict precautionary measures, experienced a feeling of loneliness and an alteration of the life-illness balance, since they were protecting a life while losing another. Conversely, previous studies (51, 52) showed how the application of these measures reduced the number of severe COPD exacerbations during the COVID-19 Pandemic. On the other hand, our results showed that as a consequence of using the mask, patients with COPD felt suffocated, with shortness of breath as if they were in a prison. The increased feeling of shortness of breath could be damaging for COPD patients, easing protective measures and increasing the risk of contagion. Tomán et al. (15) showed how for patients with COPD, dyspnea was the most terrifying symptom of progressive lung disease, and how patients tried to minimize it at all costs.

Previous studies described how patients experience denial of care, discrimination, or inequity in health care (49), and even oblivion by health authorities and health professionals (10). Our results did not show that patients with COPD have experienced lack of clinical care or inequity in access to health care. They described difficulties in accessing clinical consultations in primary care; however, similar to the rest of the people in their environment. In addition, they feel protected and safe by their healthcare professionals and by the measures taken by the health authorities to protect the population (including the most vulnerable) such as the strict confinement during the first wave of the COVID-19 outbreak for a period of 3 months (14).

Regarding vaccination, our results showed the experience of a small group of patients. However, it is paradoxical that despite the high risk of death of COPD patients in the event of contracting the SARS-CoV-2 virus and the vaccination recommendations (53), half of the participants did not wish to be vaccinated. We find these results surprising, as all the participants reported having been informed of the vaccination by their physicians and feeling very satisfied with the clinical care, trusting the professionals of the COPD unit, and confirmed having a very good professional-patient relationship. Previous studies (54, 55) reported evidence of a COVID-19 vaccine hesitancy among chronic patients. This hesitancy or distrust towards COVID-19 vaccination is not a local phenomenon but rather is widespread throughout the world (54). Factors that facilitate refusal include possible side effects, fears that the vaccine may not be safe, fear of injections, belief in natural or traditional remedies, need for more information, anti-vaccine attitudes or beliefs, believing that the vaccine will serve to those who produce this virus, i.e., conspiracy beliefs of the vaccine (55).

The qualitative design allowed us to explore and describe in depth the perspectives of patients with COPD during the COVID-19 pandemic (10, 15–19, 49). Compared to previous studies (10, 15–19), our study added these new findings: (a) The confinement measures did not cause limitation of their daily activities since they were previously limited by the illness; (b) confinement was perceived as a measure that protected them since they were more vulnerable; (c) they strictly adopted personal protection measures (masks, gloves, etc.) but they had problems adapting to the masks since they felt suffocated as if they were in a prison; (d) when walking and moving outside they tended to remove the mask to minimize the feeling of suffocation; (e) the COVID-19 vaccine was not accepted, the reasons included fear that it would further damage their lungs, the fact that the information provided by politicians was confusing and they preferred to wait getting vaccinated; (f) the professionals and the care from their reference hospital made them feel safe (unlike primary care) as they continued to monitor COPD by maintaining the in-person visit, regardless of the pandemic.

Regarding the integration of the results, the use of two analytical proposals (thematic and content) allowed us to obtain and understand the perspective of patients with COPD, as well as deepening the emotions and the tendency of polarity (acceptance/rejection) of their discourses. This did not mean that the results of both analyses coincided, but rather that they showed nuances of perspective, which only one type of analysis could not identify. Thus, in the present work, using thematic analysis, patients with COPD described important negative aspects of experiencing the first wave of the pandemic from their perspective. However, in the content analysis of his speech, through lemmatization, the polarity of his speech had a positive final result.

An important novelty of this work was the use of lemmatization as a complementary analysis proposal to other qualitative tools (such as thematic analysis), and in this way increased the credibility of the results and also the depth of the perspective analysis of the participants.

## 5 Limitations

First, regarding sample size sufficiency, previous studies (33, 34) reported that the sample size justification in qualitative health research was limited and defining sample size *a priori* is inherently problematic. Also, Sebele-Mpofu (56) describes how the definition of the concept of saturation can vary, depending on the qualitative design chosen, sampling strategy and the data collection instrument used. Due to this great variability of criteria, the authors opted for a proposal to establish the sample size based on pragmatic considerations (difficulty of access to participants due to confinement and social isolation measures) and on the information power to achieve sample size saturation (35). The inclusion of a small sample with 8 participants was in line with existing research: (a) on the sample size in qualitative studies (qualitative sample size), which described how the sample could be reached by saturation between 5–12 interviews (56–63); and (b) on qualitative studies conducted in patients with COPD during the COVID-19 pandemic, using samples of eight (15) and 10 participants (19). However, as a result of using this pragmatic consideration, along with the qualitative nature of this study, gathering the perspective of a group of COPD patients concerning a certain phenomenon and the small number of patients with COPD, this study has limitations in terms of generalizability. Finally, data collection was carried out in December 2020, time after the first wave of the COVID-19 pandemic in Spain (March 14, 2020 to June 21, 2020) (64). This could have influenced the narratives and results obtained.

## 6 Conclusion

Our results showed that COPD patients narrated how the confinement and restrictive measures of the first wave did not have a negative impact on their lives. On the contrary, fear (in all areas and situations) forced them to adopt strict protective measures to avoid contagion and impacted their daily lives. Moreover, they have experienced barriers in access to primary care, great difficulty in the use of masks, and the refusal of some patients to the COVID-19 vaccine was relevant. The content analysis showed how the main emotion was fear, and how the global polarity of the speech of patients with COPD was positive.

Future studies should analyze adherence and the use of face masks in COPD patients, and the reasons for accepting or refusing vaccination. These results may help managers to understand the impact that confinement rules and the adoption of protection measures against contagion had on the daily life of patients, as well as the importance of maintaining and the developing specialized COPD monitoring units.

## Data Availability

The original contributions presented in the study are included in the article/Supplementary material, further inquiries can be directed to the corresponding author.

## Footnotes

^1^https://www.microsoft.com/es-ww/microsoft-teams/group-chat-software
