# Supplementary material for: A novel application of lemmatize and thematic analysis to understand protective measures perspectives of patients with chronic respiratory disease during the first outbreak of COVID-19 pandemic: a qualitative study
Source: Front Public Health. 2024 Aug 29;12:1351754. doi: 10.3389/fpubh.2024.1351754 (PMC11390368; doi:10.3389/fpubh.2024.1351754)
Supplement: Supplementary file 1 [file Table_1.docx]

**Supplemental Table 1.** COREQ check list

| No | Item | Guide questions/description | Observations |
| --- | --- | --- | --- |
| **Domain 1: Research team and reflexivity** |  |  |  |
| Personal Characteristics |  |  |  |
| 1. | Interviewer/facilitator | Which author/s conducted the interview or focus group? | Page 4 |
| 2. | Credentials | What were the researcher's credentials? *E.g. PhD, MD* | Page 3 |
| 3. | Occupation | What was their occupation at the time of the study? | Page 3 |
| 4. | Gender | Was the researcher male or female? | Page 3 |
| 5. | Experience and training | What experience or training did the researcher have? | Page 3 |
| Relationship with participants |  |  |  |
| 6. | Relationship established | Was a relationship established prior to study commencement? | Page 3 |
| 7. | Participant knowledge of the interviewer | What did the participants know about the researcher? e*.g. personal goals, reasons for doing the research* | Page 3 |
| 8. | Interviewer characteristics | What characteristics were reported about the interviewer/facilitator? e.g. *Bias, assumptions, reasons and interests in the research topic* | Page 3 |
| **Domain 2: study design** |  |  |  |
| Theoretical framework |  |  |  |
| 9. | Methodological orientation and Theory | What methodological orientation was stated to underpin the study? *e.g. grounded theory, discourse analysis, ethnography, phenomenology, content analysis* | Page 2,3 |
| Participant selection |  |  |  |
| 10. | Sampling | How were participants selected? *e.g. purposive, convenience, consecutive, snowball* | Page 3 |
| 11. | Method of approach | How were participants approached? e*.g. face-to-face, telephone, mail, email* | Page 3,4 |
| 12. | Sample size | How many participants were in the study? | Page 3 |
| 13. | Non-participation | How many people refused to participate or dropped out? Reasons? | Page 3 |
| Setting |  |  |  |
| 14. | Setting of data collection | Where was the data collected? e*.g. home, clinic, workplace* | Page 3,4 |
| 15. | Presence of non-participants | Was anyone else present besides the participants and researchers? | Page 3,4 |
| 16. | Description of sample | What are the important characteristics of the sample? *e.g. demographic data, date* | Page 3,5, table 4 |
| Data collection |  |  |  |
| 17. | Interview guide | Were questions, prompts, guides provided by the authors? Was it pilot tested? | Page 3,4, table 1, table 2 |
| 18. | Repeat interviews | Were repeat interviews carried out? If yes, how many? | Page 3,4, table 1, table 2 |
| 19. | Audio/visual recording | Did the research use audio or visual recording to collect the data? | Page 3,4, table 1, table 2 |
| 20. | Field notes | Were field notes made during and/or after the interview or focus group? | Page 3,4, table 1, table 2 |
| 21. | Duration | What was the duration of the interviews or focus group? | Page 3,4, table 1, table 2 |
| 22. | Data saturation | Was data saturation discussed? | Page 3, page 11 |
| 23. | Transcripts returned | Were transcripts returned to participants for comment and/or correction? | Page 5, table 3 |
| **Domain 3: analysis and findings**z |  |  |  |
| Data analysis |  |  |  |
| 24. | Number of data coders | How many data coders coded the data? | Page 4, 5, figure 1, table S3 |
| 25. | Description of the coding tree | Did authors provide a description of the coding tree? | Page 4, 5, figure 1, table S3 |
| 26. | Derivation of themes | Were themes identified in advance or derived from the data? | Page 4, 5, figure 1, table S3 |
| 27. | Software | What software, if applicable, was used to manage the data? | Page 4, 5, figure 1, table S3 |
| 28. | Participant checking | Did participants provide feedback on the findings? | Page 5, table 3 |
| Reporting |  |  |  |
| 29. | Quotations presented | Were participant quotations presented to illustrate the themes / findings? Was each quotation identified? e*.g. participant number* | Page 5-10, table 4, figure 2, figure 3, figure 4 |
| 30. | Data and findings consistent | Was there consistency between the data presented and the findings? | Page 5-10, table 4, figure 2, figure 3, figure 4 |
| 31. | Clarity of major themes | Were major themes clearly presented in the findings? | Page 5-10, table 4, figure 2, figure 3, figure 4 |
| 32. | Clarity of minor themes | Is there a description of diverse cases or discussion of minor themes? | Page 5-10, table 4, figure 2, figure 3, figure 4 |

**Supplemental Table 2.** SRQR check list

| No | Item | Guide questions/description | Observations |
| --- | --- | --- | --- |
|  | **Title and abstract** |  |  |
| S1 | Title | Concise description of the nature and topic of the study Identifying the study as qualitative or indicating the approach (e.g., ethnography, grounded theory) or data collection methods (e.g., interview, focus group) is recommended | Page 1 |
| S2 | Abstract | Summary of key elements of the study using the abstract format of the intended publication; typically includes background, purpose, methods, results, and conclusions | Page 1-2 |
|  | **Introduction** |  |  |
| S3 | Problem formulation | Description and significance of the problem/phenomenon studied; review of relevant theory and empirical work; problem statement | Page 2 |
| S4 | Purpose or research question | Purpose of the study and specific objectives or questions | Page 2 |
|  | **Methods** |  |  |
| S5 | Qualitative approach and research paradigm | Qualitative approach (e.g., ethnography, grounded theory, case study, phenomenology, narrative research) and guiding theory if appropriate; identifying the research paradigm (e.g., postpositivist, constructivist/interpretivist) is also recommended; rationale | Page 2,3 |
| S6 | Researcher characteristics and reflexivity | Researchers’ characteristics that may influence the research, including personal attributes, qualifications/experience, relationship with participants, assumptions, and/or presuppositions; potential or actual interaction between researchers’ characteristics and the research questions, approach, methods, results, and/or transferability | Page 3 |
| S7 | Context | Setting/site and salient contextual factors; rationale | Page 2 |
| S8 | Sampling strategy | How and why research participants, documents, or events were selected; criteria for deciding when no further sampling was necessary (e.g., sampling saturation); rationale | Page 3 |
| S9 | Ethical issues pertaining to human subjects | Documentation of approval by an appropriate ethics review board and participant consent, or explanation for lack thereof; other confidentiality and data security issues | Page 5 |
| S10 | Data collection methods | Types of data collected; details of data collection procedures including (as appropriate) start and stop dates of data collection and analysis, iterative process, triangulation of sources/methods, and modification of procedures in response to evolving study findings; rationale | Page 3,4, table 1, table 2 |
| S11 | Data collection instruments and technologies | Description of instruments (e.g., interview guides, questionnaires) and devices (e.g., audio recorders) used for data collection; if/how the instrument(s) changed over the course of the study | Page 3,4, table 1, table 2 |
| S12 | Units of study | Number and relevant characteristics of participants, documents, or events included in the study; level of participation (could be reported in results) | Page 3,5, table 4 |
| S13 | Data processing | Methods for processing data prior to and during analysis, including transcription, data entry, data management and security, verification of data integrity, data coding, and anonymization/deidentification of excerpts | Page 4,5, Table S3, Figure 1 |
| S14 | Data analysis | Process by which inferences, themes, etc., were identified and developed, including the researchers involved in data analysis; usually references a specific paradigm or approach; rationale | Page 4,5, Table S3, Figure 1 |
| S15 | Techniques to enhance trustworthiness | Techniques to enhance trustworthiness and credibility of data analysis (e.g., member checking, audit trail, triangulation); rationale | Page 5, table 3 |
|  | **Results/Findings** |  |  |
| S16 | Synthesis and interpretation | Main findings (e.g., interpretations, inferences, and themes); might include development of a theory or model, or integration with prior research or theory | Page 5-10, table 4, figure 2, figure 3, figure 4 |
| S17 | Links to empirical data | Evidence (e.g., quotes, field notes, text excerpts, photographs) to substantiate analytic findings | Page 5-10, table 4, figure 2, figure 3, figure 4 |
|  | **Discusssion** |  |  |
| S18 | Integration with prior work, implications,  transferability, and contribution(s) to the field | Short summary of main findings; explanation of how findings and conclusions connect to, support, elaborate on, or challenge conclusions of earlier scholarship; discussion of scope of application/ generalizability; identification of unique contribution(s) to scholarship in a discipline or field | Page 10-11 |
| S19 | Limitations | Trustworthiness and limitations of findings | Page 11 |
|  | **Other** |  |  |
| S20 | Conflicts of interest | Potential sources of influence or perceived influence on study conduct and conclusions; how these were managed | None |
| S21 | Funding | Sources of funding and other support; role of funders in data collection, interpretation, and reporting | This research was funded by a grant from Sociedad Española de Neumología y Cirugía Torácica (SEPAR) [Spanish Society of Pneumology and Thoracic Surgery] (SEPAR Research Grants, 2020-2021). The SEPAR has not been specifically involved in the research. |

**Supplemental Table 3.** Formula and dictionaries used to calculate polarity

| To calculate the polarity (δ), a context cluster of words (x^T^_i_) was formed around each polarized word using the Bing dictionary (2010), taking, by default, four words before and two words after it (if there is any comma in the cluster, it will only include the words that are after the comma) and this will be treated as valence shifters.  The words in this cluster are labeled as neutral (x^0^_i_), negators (x^N^_i_), amplifiers (x^a^_i_) or de-amplifiers (x^d^_i_) using the dictionary SODictionariesV1.11Spa2 and the negators proposed by Vilares et al. (2013). Neutral words do not add to the equation but affect the word count (n).  Each polarized word (negative or positive) is weighted (w) based on the context cluster weights (x^T^_i_) and further weighted by the number and position of the valence shifters directly surrounding it. A weight (c) can be added and applied to both amplifiers and de-amplifiers (with a default value of 0.8 and a lower limit for the de-amplifiers of -1).  Finally, the context cluster (x^T^_i_) is added and divided by the square root of the number of words (√n) to generate a polarity score (δ) that by default is not limited in value. |
| --- |
| The end result is the formula:  δ = x^T^_i_ / √n |
| Where:  x^T^_i_ = ∑ ((1 + c(x^A^_i_ - x^A^_i_)) ^.^ w(-1) ^∑xNi^)  x^A^_i_ = ∑ (w_neg_ ^.^ x^a^_i_)  x^D^_i_ = max (x^D´^_i_ - 1)  x^D´^_i_ = ∑ (-w_neg_ ^.^ x^a^_i_ + x^d^_i_)  w_neg_ = (∑x^N^_i_) |
